# Bibliometric analysis of exercise and cancer prognosis research: trends, thematic evolution, and global collaborations (2015–2024)

**DOI:** 10.3389/fonc.2026.1710464

**Published:** 2026-06-02

**Authors:** Xiaodong Wang, Di Xiong, Qianqian Wang, Yiping Huang, Gouping Ding, Yixuan Tang, Songli Cui

**Affiliations:** 1 Department of Oncology, Zhuzhou Hospital Affiliated to Xiangya School of Medicine, Central South University, Zhuzhou, China; 2 Department of General Medicine, Zhuzhou Hospital Affiliated to Xiangya School of Medicine, Central South University, Zhuzhou, China

**Keywords:** bibliometric analysis, cancer prognosis, exercise oncology, physical activity, research trends

## Abstract

**Background:**

Physical activity and structured exercise are increasingly recognized as modifiable factors that influence cancer prognosis, recurrence risk, and quality of life. Despite rapid growth in this literature, the structure, thematic evolution, and international distribution of research linking exercise to cancer prognosis have not been systematically mapped.

**Methods:**

We conducted a bibliometric analysis of publications indexed in the Web of Science Core Collection and Scopus on 22 August 2025. Search terms combined exercise/physical activity with cancer prognosis, survival, or recurrence. After deduplication, 3,261 unique English-language articles and reviews (2015–2024) were analyzed using the bibliometrix R package, VOSviewer, and CiteSpace for publication trends, co-authorship networks, keyword co-occurrence, and citation b ursts.

**Results:**

Annual output nearly doubled, rising from 211 publications in 2015 to 483 in 2024. The United States (n = 850) and China (n = 780) led global production, jointly accounting for approximately 50% of all papers. Keyword co-occurrence analysis identified five major thematic clusters: clinical rehabilitation and quality-of-life outcomes, biological mechanisms (including muscle physiology and sarcopenia), epidemiology and risk factors, cancer survivorship (emphasizing fatigue and psychosocial domains), and exercise interventions during active treatment. Citation patterns showed heavy reliance on foundational oncology guidelines and reviews from adjacent fields.

**Conclusions:**

Research on exercise and cancer prognosis has expanded rapidly and consolidated thematically, with growing emphasis on personalized prescriptions and rigorous trial evidence. Output remains concentrated in high-income countries, highlighting the need for broader international collaboration and capacity building to support equitable translation into multidisciplinary cancer care.

## Graphical Abstract

**Figure:**
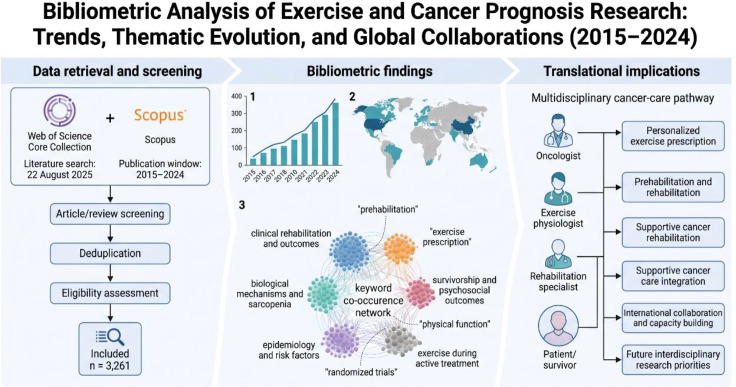

## 1 Introduction

Cancer remains a leading cause of morbidity and mortality worldwide, with an estimated 19.3 million new cases and nearly 10 million deaths in 2020 (1, 2). Although advances in early detection and multimodal therapy have improved survival for many malignancies, long-term prognosis continues to be shaped by tumor biology, treatment sequelae, and modifiable patient factors. Among these, physical activity and structured exercise have emerged as critical determinants of clinical outcomes in cancer patients and survivors. Accumulating evidence from large cohort studies and meta-analyses links higher post-diagnosis physical activity levels to reduced risks of recurrence and improved overall and cancer-specific survival across several tumor types (3–5). These benefits are attributed to multiple mechanisms, including enhanced cardiopulmonary fitness, modulation of systemic inflammation and immune surveillance, preservation of skeletal muscle mass, and improved treatment tolerance (6, 7).

The field of exercise oncology has therefore expanded rapidly over the past decade. Landmark randomized trials, such as the CHALLENGE trial in colon cancer survivors, together with international guidelines endorsing exercise as standard supportive care, have reinforced its prognostic relevance (8–10). Research has diversified to encompass mechanistic investigations of sarcopenia and metabolism, exercise prescription during active treatment, and implementation strategies in survivorship care (6, 11). This growth has generated a voluminous and heterogeneous body of literature that now spans clinical outcomes, biological pathways, epidemiological associations, and health-services research.

Despite this momentum, the overall structure and evolution of the exercise–prognosis literature have not been comprehensively characterized. Although the broader field of exercise oncology has received increasing attention in recent years, a dedicated bibliometric and scientometric analysis focused on prognosis-related outcomes during the contemporary period (2015–2024) is lacking. Such an evaluation can objectively delineate temporal trends in publication volume, map international collaboration networks, identify dominant thematic clusters through keyword co-occurrence, and detect shifts in research emphasis via citation bursts. These insights are essential for highlighting productive regions, emerging priorities, and persistent gaps—particularly the under-representation of low- and middle-income settings.

The present study therefore applied bibliometric and scientometric methods to publications indexed in the Web of Science Core Collection and Scopus from 2015 through 2024. By integrating quantitative indicators of output, network visualizations of co-authorship and keywords, and citation dynamics, we aimed to provide a clear overview of the global research landscape on exercise and cancer prognosis. The findings are intended to inform future investigative priorities, facilitate cross-disciplinary and international collaboration, and support the evidence-based integration of exercise into multidisciplinary cancer care.

## 2 Methods

### 2.1 Data sources and search strategy

**Figure 1:**
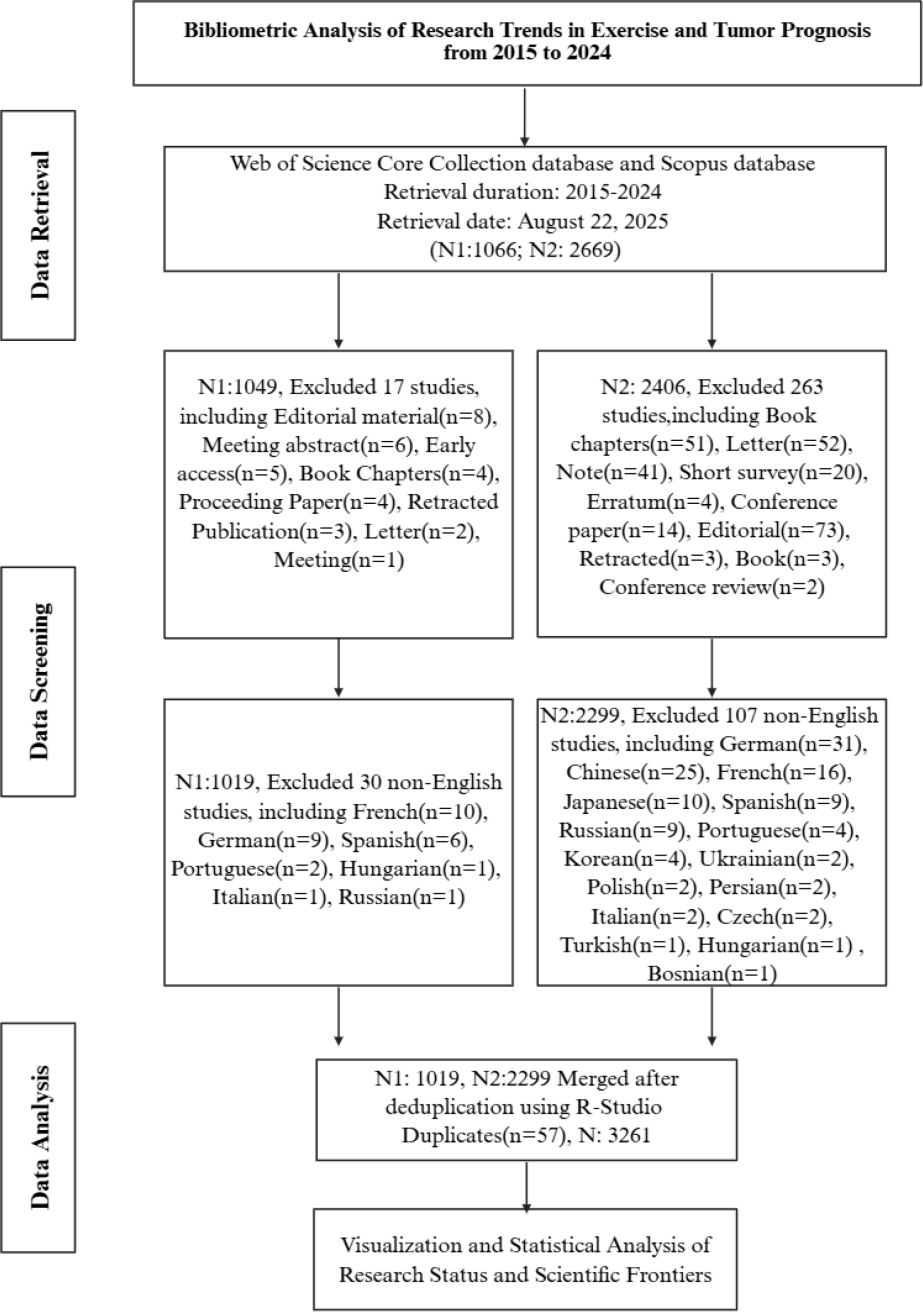
Study flow diagram for literature identification, screening, and inclusion. Records were retrieved from the Web of Science Core Collection and Scopus on 22 August 2025 for publications issued between 2015 and 2024 using terms related to exercise/physical activity, cancer, and prognosis. After removal of duplicate records, titles and abstracts were screened independently by two reviewers according to predefined eligibility criteria; non-article literature and records outside the study scope were excluded. The final merged dataset comprised 3,261 publications included in the bibliometric analyses.

We conducted a literature search in the Web of Science Core Collection and Scopus on 22 August 2025. The search strategy integrated terms related to exercise (such as exercise and physical activity) with those related to cancer prognosis (such as survival, recurrence, and prognosis). Full search queries appear in **Supplementary Appendix S2**. Peer-reviewed journal articles and reviews published between 2015 and 2024 were included regardless of language. Records retrieved from both databases were merged and duplicate entries removed. The final dataset comprised 3261 unique publications (**Figure 1**).

### 2.2 Bibliometric analysis

All bibliometric indicators, including publication counts and citation frequencies, were calculated from the merged dataset. General metrics were computed with the bibliometrix R package (12, 13). Co-authorship and keyword co-occurrence networks were constructed and visualized using VOSviewer version 1.6.18 (14, 15). CiteSpace version 5.8.R3 served to detect citation bursts (16–18). In VOSviewer, the keyword co-occurrence network was generated from author keywords and index terms, with a minimum occurrence threshold of 40 applied to retain high-frequency terms. Clusters were identified with the default resolution parameter and labeled thematically. Detailed parameters, including thresholds and clustering resolution, are specified in the figure legends.

### 2.3 Data preprocessing and normalization

Raw data were cleaned prior to analysis to improve consistency. Duplicate records from overlapping Web of Science and Scopus coverage were eliminated. Author names and affiliations were standardized by merging variants. Keywords and index terms were reviewed, and synonyms together with spelling variants were consolidated to reduce redundancy. For example, exercise and physical activity were treated equivalently, and singular and plural forms were unified. Standardization was performed through manual curation without a specialized thesaurus. These steps ensured that co-occurrence networks accurately reflected conceptual relationships rather than labeling inconsistencies.

## 3 Results

### 3.1 Publication trends and collaboration

**Figure 2:**
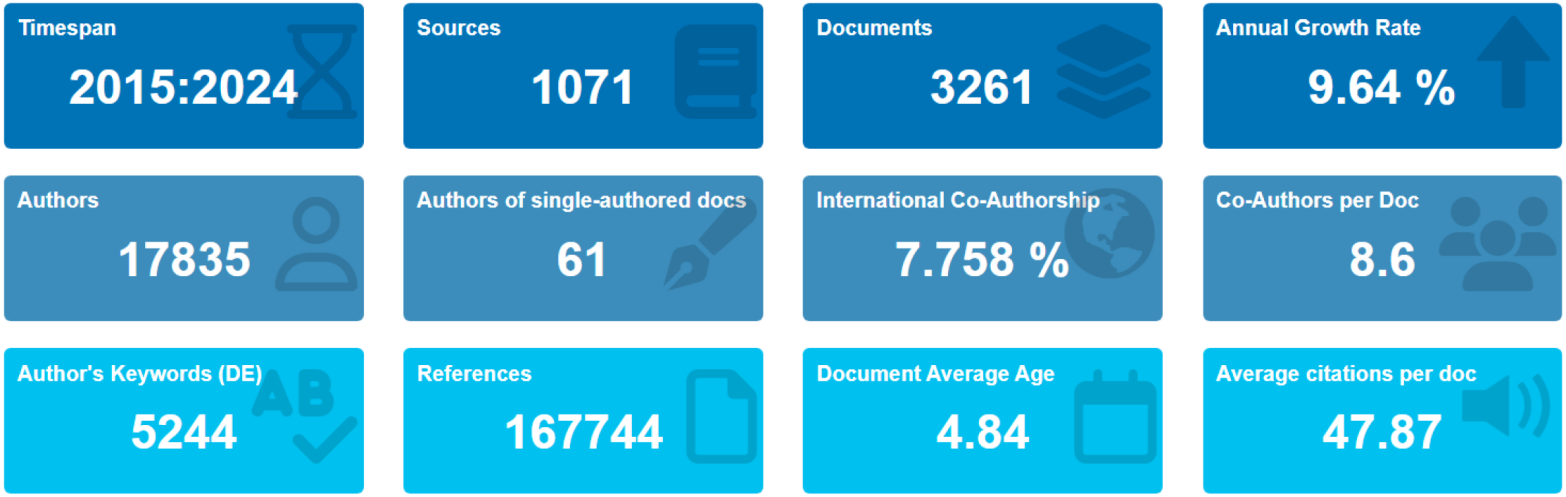
Bibliometric overview of the exercise–cancer prognosis literature (2015–2024). The dashboard summarizes the main descriptive indicators for the final corpus of 3,261 publications, including timespan, number of sources, annual growth rate, number of authors, proportion of single-authored documents, international co-authorship, co-authors per document, author keywords, total cited references, mean document age, and average citations per document. Metrics were generated using the bibliometrix package in R and are presented to provide a high-level overview of field productivity, collaboration, and citation characteristics.

**Figure 3:**
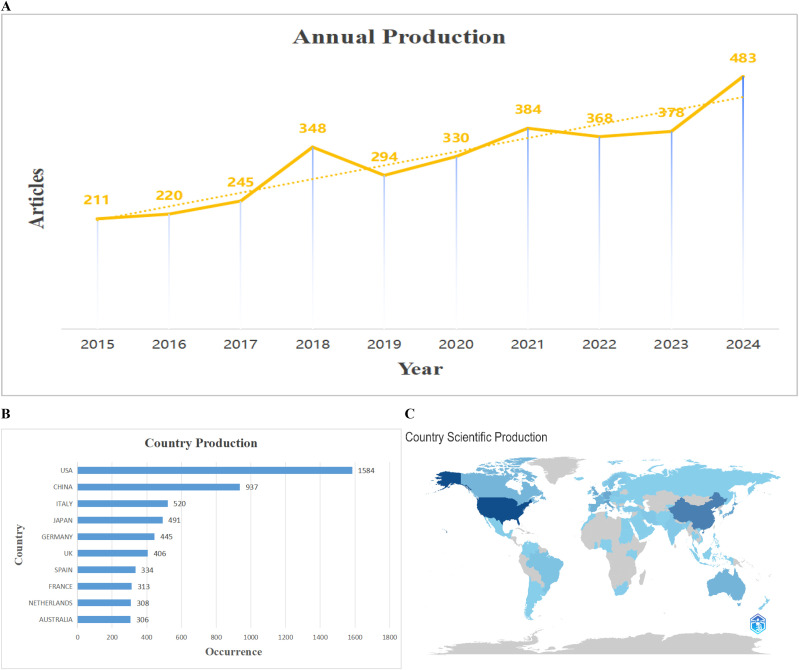
Annual publication trend and geographic distribution of research on exercise and cancer prognosis (2015–2024). **(A)** Annual number of included publications, with a fitted trend line indicating the overall growth of the field during the study period. **(B)** Top 10 countries ranked by publication output in the merged dataset. **(C)** Global scientific production displayed as a choropleth map, in which darker shading denotes greater publication output. Country-level production reflects author-affiliation data in the retrieved records and illustrates the geographic concentration of research activity.

Annual publications addressing exercise and cancer prognosis rose substantially from 211 in 2015 to 483 in 2024, reflecting markedly increased research interest. **Figure 2** summarizes key bibliometric trends. The median number of authors per paper increased from 4 to 6, while the body of work expanded across a broader range of interdisciplinary journals. The United States (n = 850) and China (n = 780) were the leading contributors, jointly accounting for approximately 50% of all publications. Multinational authorship was relatively uncommon, occurring in only 7.8% of publications. Overall output remained highly concentrated: the top 10 countries produced nearly 90% of papers, with limited contributions from low- and middle-income regions. Country-level publication counts are displayed in **Figure 3**.

### 3.2 Keyword co-occurrence and thematic clusters

**Figure 4:**
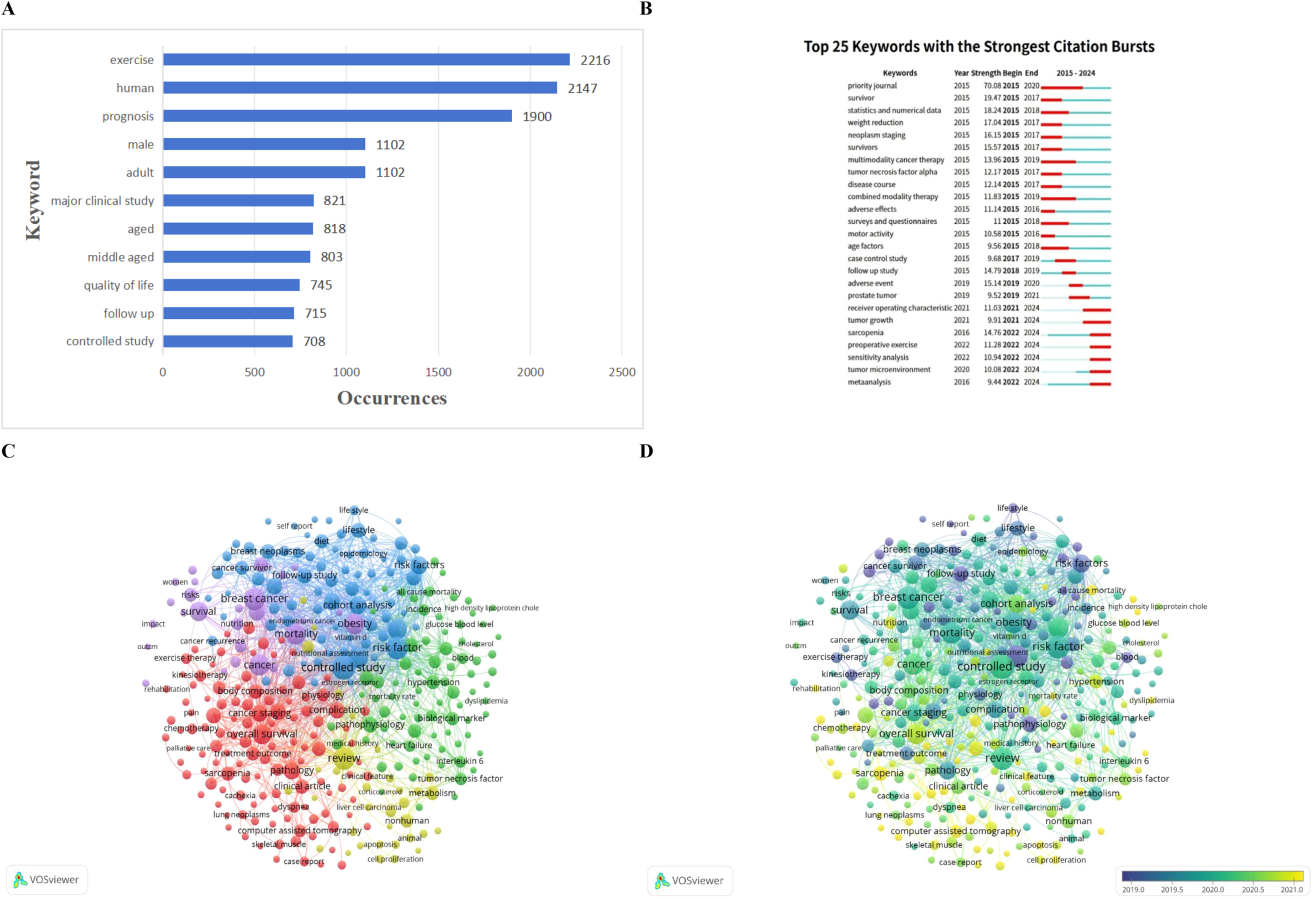
Keyword structure, burst dynamics, and temporal evolution of research on exercise and cancer prognosis (2015–2024). **(A)** Top 10 keywords ranked by occurrence frequency in the corpus. **(B)** Top 25 keywords with the strongest citation bursts detected by CiteSpace; burst strength and active burst intervals are shown, and red segments indicate the burst periods. **(C)** Keyword co-occurrence network generated in VOSviewer from standardized author keywords and index terms after manual preprocessing and synonym harmonization. Only terms with a minimum occurrence of 40 were included in the network. Node size represents keyword frequency, line thickness represents co-occurrence strength, and colors denote clusters identified using VOSviewer’s default clustering settings; cluster labels were assigned manually according to the dominant terms in each group. **(D)** Overlay visualization of the same keyword network colored by average publication year, with cooler colors indicating earlier average occurrence and warmer colors indicating more recent average occurrence. The thematic clusters represent semantic proximity and shared research attention rather than direct causal, mechanistic, or clinical relationships.

Analysis of author keywords and index terms in VOSviewer, using a minimum occurrence threshold of 40, revealed five major thematic clusters (**Figure 4**; **Supplementary Appendix S1**). Cluster 1 (red; 119 items) centered on clinical oncology and rehabilitation outcomes, incorporating topics such as exercise training, cardiopulmonary function, and quality of life. Cluster 2 (green; 92 items) focused on biological mechanisms, with emphasis on muscle physiology, metabolism, and sarcopenia. Cluster 3 (blue; 78 items) addressed epidemiology and risk factors, including cancer incidence, obesity, and inflammation. Cluster 4 (purple; 32 items) concerned cancer survivorship and quality of life, particularly fatigue and psychosocial outcomes. Cluster 5 (yellow; 26 items) examined exercise interventions during active treatment, encompassing chemotherapy, postoperative rehabilitation, and supportive care. Comprehensive keyword lists for each cluster are provided in **Supplementary Appendix S1**.

Collectively, these clusters indicate that the literature is thematically consolidated around clinical outcomes, mechanistic research, and survivorship issues, with substantial overlap between rehabilitation-focused and treatment-related topics. Temporal keyword trends revealed an evolution in research emphasis. From 2015 to 2019, general terms such as exercise oncology and quality of life predominated. In contrast, the period 2020–2024 saw strong citation bursts for more specific terms including exercise prescription, physical function, and randomized controlled trial, suggesting growing interest in personalized exercise interventions and rigorous clinical evidence.

### 3.3 Citation analysis

**Figure 5:**
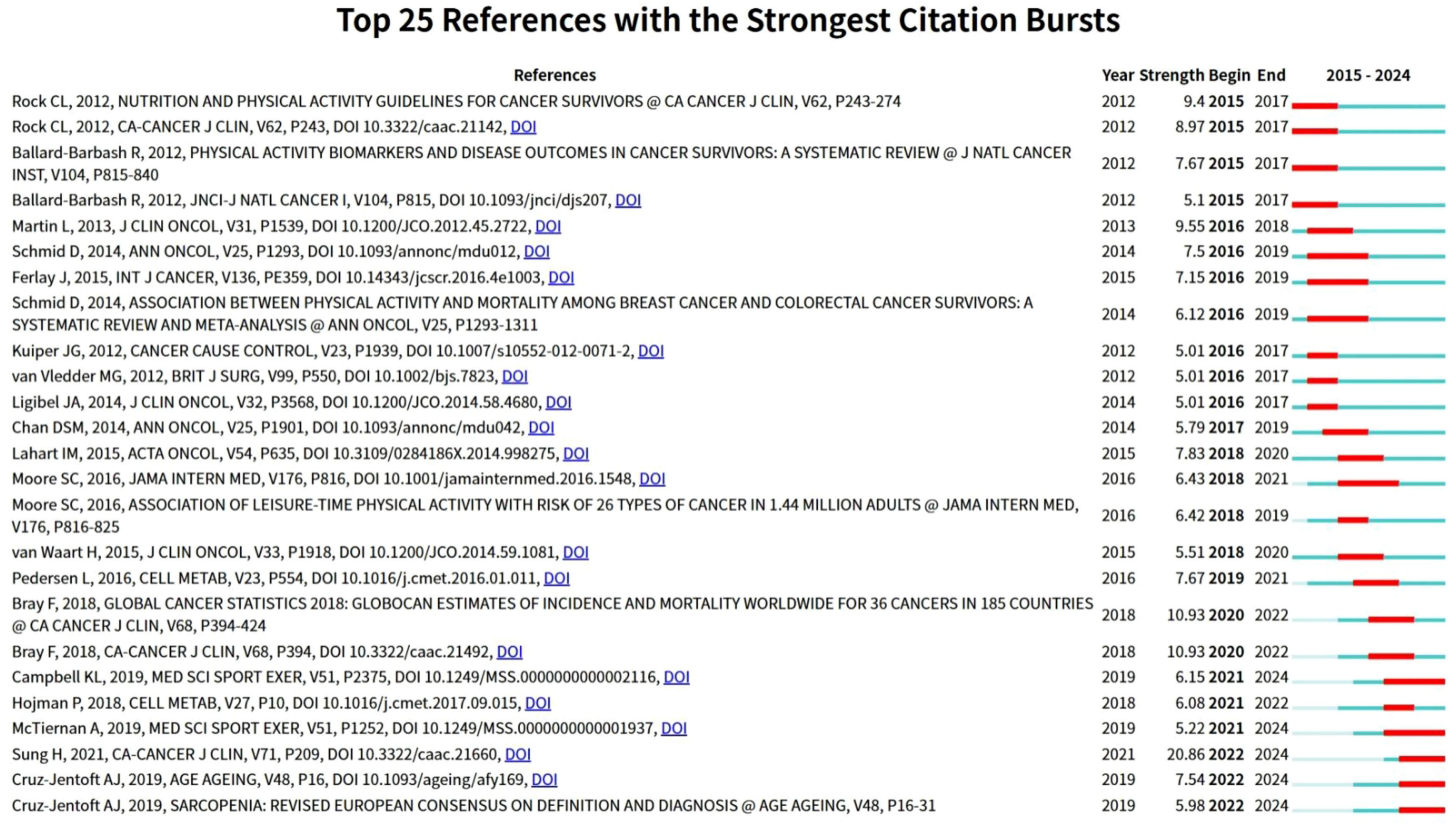
References with the strongest citation bursts within the exercise–cancer prognosis corpus (2015–2024). The figure presents the top 25 cited references exhibiting the strongest citation bursts as detected by CiteSpace, together with bibliographic information, burst strength, and burst interval. Red bars indicate the periods during which a reference experienced a marked increase in attention, whereas the blue baseline represents the full study timeline. These burst patterns reflect shifts in influence within the analyzed corpus and may include broadly cited guidelines or reviews from adjacent disciplines in addition to exercise-oncology-specific publications.

Citation patterns indicated that the knowledge base of this field is strongly interdisciplinary. As shown in **Table 1**, the most highly cited references associated with the dataset were dominated by broadly influential clinical guidelines and review articles from general medicine and related specialties, rather than by exercise-oncology prognosis studies alone. Several top-ranked references were high-impact guidelines or disease overviews published in major journals, suggesting that research on exercise and cancer prognosis is embedded within a wider body of oncology, rehabilitation, and chronic disease literature. **Figure 5** provides a complementary perspective by identifying the references with the strongest citation bursts within the corpus. Unlike total citation counts, citation bursts reflect periods of rapidly increasing attention and therefore capture temporal shifts in influence rather than overall citation volume. Taken together, these findings suggest that the citation structure of this field is anchored by authoritative cross-disciplinary publications, while the burst analysis highlights references that became especially prominent during specific periods of development. Overall, the literature on exercise and cancer prognosis appears to rely not only on exercise-specific evidence, but also on broader supportive-care, survivorship, and disease-oriented scholarship.

**Table 1:** Top 10 highly cited publications in the dataset on exercise and tumor prognosis (2015–2024), ranked by total citations.

Rank	Title	Publication year	Journal	JCR category	First author	Total citations
1	2016 ESC Guidelines for the diagnosis and treatment of acute and chronic heart failure: The Task Force for the diagnosis and treatment of acute and chronic heart failure of the European Society of Cardiology (ESC)Developed with the special contribution of the Heart Failure Association (HFA) of the ESC (19)	2016	European Heart Journal	Cardiac & Cardiovascular Systems (Q1)	Piotr Ponikowski	11136
2	2016 ESC Guidelines for the diagnosis and treatment of acute and chronic heart failure: The Task Force for the diagnosis and treatment of acute and chronic heart failure of the European Society of Cardiology (ESC). Developed with the special contribution of the Heart Failure Association (HFA) of the ESC (19)	2016	European Journal of Heart Failure	Cardiac & Cardiovascular Systems (Q1)	Piotr Ponikowski	5673
3	Colorectal cancer (20)	2019	Lancet	Medicine, General & Internal (Q1)	Evelien Dekker	3602
4	What low back pain is and why we need to pay attention (21)	2018	Lancet	Medicine, General & Internal (Q1)	Jan Hartvigsen	2849
5	2022 ESC/ERS Guidelines for the diagnosis and treatment of pulmonary hypertension (22)	2022	European Heart Journal	Cardiac & Cardiovascular Systems (Q1)	Marc Humbert	2498
6	2023 ESC Guidelines for the management of acute coronary syndromes (23)	2023	European Heart Journal	Cardiac & Cardiovascular Systems (Q1)	Robert A Byrne	2398
7	2018 ESC/ESH Guidelines for the management of arterial hypertension: The Task Force for the management of arterial hypertension of the European Society of Cardiology and the European Society of Hypertension: The Task Force for the management of arterial hypertension of the European Society of Cardiology and the European Society of Hypertension (24)	2018	Journal of Hypertension	Peripheral Vascular Disease (Q1)	Bryan Williams	2352
8	Diagnosis and Treatment of Parkinson Disease: A Review (25)	2020	JAMA - Journal of the American Medical Association	Medicine, General & Internal (Q1)	Melissa J Armstrong	2107
9	Renal cell carcinoma (26)	2017	Nature Reviews Disease Primers	Medicine, General & Internal (Q1)	James J Hsieh	2058
10	Nonalcoholic fatty liver disease: a systematic review (27)	2015	JAMA - Journal of the American Medical Association	Medicine, General & Internal (Q1)	Mary E Rinella	181.18

This table describes citation prominence within the knowledge base surrounding the analyzed corpus. The total citation values reflect database-indexed citation accumulation and are therefore influenced by a publication’s visibility across the broader biomedical literature. As a result, high-impact clinical guidelines and general review articles from adjacent fields may rank highly even when they are not specific to exercise-oncology prognosis research. Accordingly, this table should not be interpreted as a definitive list of landmark exercise-oncology prognosis trials. JCR, Journal Citation Reports; Q1, first quartile.

## 4 Discussion

### 4.1 Global output and collaboration

This analysis demonstrates a rapid expansion in research on exercise and cancer prognosis from 2015 to 2024, with annual publication numbers nearly doubling during this period. The observed growth aligns with previous bibliometric studies documenting similar upward trends in the broader exercise oncology domain. The United States and China were the most productive countries, jointly accounting for approximately half of all publications. This dominance reflects their substantial research infrastructure and funding support. However, global output remained markedly concentrated, with the top 10 countries responsible for nearly 90% of publications and limited representation from low- and middle-income regions. These geographic imbalances highlight the need for greater international engagement and capacity building to incorporate more diverse clinical and cultural perspectives into the field.

### 4.2 Thematic consolidation and evolution

Keyword co-occurrence analysis identified five major thematic clusters, indicating that research on exercise and cancer prognosis has become increasingly consolidated. The largest cluster focused on clinical oncology and rehabilitation outcomes, including exercise training, cardiopulmonary function, and quality of life. The remaining clusters addressed biological mechanisms (with emphasis on muscle physiology, metabolism, and sarcopenia), epidemiology and risk factors, cancer survivorship with attention to fatigue and psychosocial outcomes, and exercise interventions during active treatment phases. Temporal analysis revealed a clear evolution in research focus. Earlier years were characterized by general terms such as exercise oncology and quality of life, whereas more recent literature showed strong citation bursts for specific concepts including exercise prescription, physical function, and randomized controlled trials. These shifts suggest growing emphasis on personalized and rigorously tested interventions.

These thematic clusters should be interpreted with appropriate caution. Co-occurrence patterns indicate prevailing research interests and conceptual associations rather than direct causal relationships. For instance, the frequent co-occurrence of skeletal muscle and cancer prognosis terms signals active scientific interest but does not establish that exercise modulates survival exclusively through muscle-mediated pathways. Detailed keyword lists for each cluster are provided in **Supplementary Appendix S1**.

### 4.3 Citation patterns and influences

Citation analysis revealed that the field of exercise and cancer prognosis draws substantially from clinical guidelines, systematic reviews, and influential studies from adjacent disciplines within oncology. Many of the most frequently cited references and those displaying the strongest citation bursts originated from broader cancer research, including foundational work on cancer cachexia and supportive care. This pattern underscores the interdisciplinary nature of exercise oncology and its continued reliance on established knowledge generated outside narrowly defined exercise-specific studies.

### 4.4 Methodological considerations and limitations

A minimum occurrence threshold of 40 was applied when constructing the keyword co-occurrence network. This threshold was chosen to achieve an effective balance between network interpretability and comprehensive coverage of core concepts. We recognize that different thresholds would influence both the size and the thematic composition of the resulting clusters.

The present study has several limitations. The literature search was confined to the Web of Science Core Collection and Scopus, which may have led to underrepresentation of research indexed only in other databases or published in non-English languages. Additionally, the analysis was primarily descriptive in nature. Advanced network metrics, such as betweenness centrality and total link strength, were not calculated. Incorporating these quantitative indicators in future scientometric investigations would provide deeper insights into the dynamics of scientific collaboration and knowledge flow within the field.

### 4.5 Translational and practical implications

The findings have several translational and practical implications. The strong focus on functional outcomes and survival metrics supports the systematic integration of structured exercise programs into multidisciplinary cancer care, including prehabilitation before surgery and rehabilitation during and after treatment. The recent emergence of terms related to exercise prescription and digital health technologies indicates a promising direction toward more individualized and scalable interventions. To fully realize these opportunities, enhanced collaboration between oncologists, exercise physiologists, rehabilitation specialists, and behavioral scientists will be essential. Finally, the pronounced concentration of research activity in high-income countries emphasizes the importance of targeted capacity-building efforts in underrepresented regions. Such initiatives are critical to ensure that advances in exercise-based oncology care are both globally relevant and equitably accessible.

## 5 Conclusions

In conclusion, this bibliometric analysis provides a detailed overview of the structure, growth, and thematic evolution of research on exercise and cancer prognosis between 2015 and 2024. The field has grown rapidly, achieved notable thematic consolidation, and remains led by a relatively small number of countries. These insights can inform future research priorities, facilitate international collaborations, and support the development of evidence-based clinical guidelines. Subsequent studies would benefit from expanded data sources and the application of more advanced network analysis techniques to further advance understanding in this important area of cancer research.

## Data Availability

Publicly available datasets were analyzed in this study. The bibliometric data analyzed in this study were retrieved from the Web of Science Core Collection (Science Citation Index Expanded) and Scopus databases, as detailed in the Materials and Methods section (Section 2.1). Raw search results, deduplicated datasets, and merged files generated using R (v4.5.1) with the bibliometrix package are available from the corresponding author upon reasonable request. All analyses were conducted using publicly accessible tools including VOSviewer (v1.6.20) and CiteSpace (v6.4.R1), with no proprietary data involved.
