# Supplementary material for: Bibliometric analysis of exercise and cancer prognosis research: trends, thematic evolution, and global collaborations (2015–2024)
Source: Front Oncol. 2026 Jun 2;16:1710464. doi: 10.3389/fonc.2026.1710464 (PMC13269046; doi:10.3389/fonc.2026.1710464)
Supplement: Supplementary file 1 [file DataSheet1.docx]

**Supplementary Appendix**

**Appendix S1. Keyword co-occurrence clusters (VOSviewer)**

Note: Keywords were extracted from the VOSviewer keyword co-occurrence network (minimum occurrences = 50; 334 keywords; 5 clusters). Cluster labels were assigned manually based on dominant high-frequency terms (corresponding to Fig. 5C in the main text).

**Cluster 1 (119 items): Clinical oncology / treatment context / functional & rehabilitation outcomes**

adjuvant chemotherapy; advanced cancer; adverse event; aerobic exercise; anemia; antineoplastic agent; anxiety; body composition; body weight loss; cachexia; cancer chemotherapy; cancer immunotherapy; cancer patient; cancer radiotherapy; cancer staging; cancer surgery; cancer therapy; carboplatin; cardiopulmonary exercise; cardiorespiratory fitness; case report; chemoradiotherapy; chemotherapy; child; cisplatin; clinical article; clinical assessment; clinical effectiveness; clinical feature; clinical outcome; clinical practice; combined modality therapy; complication; computer assisted tomography; cyclophosphamide; daily life activity; depression; diagnostic imaging; diet therapy; doxorubicin; dyspnea; esophagus cancer; exercise test; exercise therapy; exercise tolerance; fatigue; fitness; fluorouracil; forced expiratory volume; frailty; functional status; grip strength; head and neck cancer; health status; hemoglobin; histology; histopathology; human tissue; immunohistochemistry; kinesiotherapy; length of stay; lung cancer; lung neoplasms; lung tumor; malnutrition; medical history; multimodality cancer therapy; muscle atrophy; muscle mass; muscle strength; muscle, skeletal; neoadjuvant chemotherapy; non-small cell lung cancer; nuclear magnetic resonance; nutritional status; oncology; outcome assessment; overall survival; oxygen consumption; paclitaxel; pain; palliative care; palliative therapy; pancreas cancer; pathology; patient care; patient compliance; patient-reported outcome; physical examination; physical performance; physiology; physiotherapy; pilot study; postoperative complication; postoperative period; practice guideline; procedures; progression-free survival; radiation; randomized controlled trial; rehabilitation; resistance training; retrospective study; sarcopenia; scoring system; six-minute walk test; skeletal muscle; stomach cancer; surgery; therapy; thorax radiography; treatment duration; treatment outcome; treatment response; walking; x-ray computed tomography.

**Cluster 2 (80 items): Cardiometabolic / inflammation / comorbidity & risk markers**

acetylsalicylic acid; adiponectin; aging; albumin; all-cause mortality; anthropometry; atrial fibrillation; beta-adrenergic receptor; biological marker; biomarkers; blood; blood pressure; C-reactive protein; cardiovascular disease; cardiovascular mortality; cardiovascular risk; cause of death; cerebrovascular accident; cholesterol; chronic disease; chronic obstructive lung disease; comorbidity; coronary artery disease; corticosteroid; creatinine; cytokine; diabetes mellitus; diagnostic test accuracy; diastolic blood pressure; dipeptidyl carboxypeptidase; disease association; disease severity; dyslipidemia; echocardiography; enzyme-linked immunoassay; glucose; glucose blood level; heart failure; heart infarction; heart rate; hemoglobin A1c; high-density lipoprotein; high-risk patient; hospitalization; hydroxymethylglutaryl-CoA reductase; hypertension; incidence; inflammation; insulin; insulin resistance; interleukin-1beta; interleukin-6; ischemic heart disease; leptin; low-density lipoprotein; malignant neoplasm; metabolic syndrome; metformin; morbidity; mortality rate; mortality risk; non-insulin-dependent; nonsteroidal anti-inflammatory; pathogenesis; pathophysiology; prediction; predictive value; prevalence; protein blood level; receiver operating characteristic; risk assessment; sensitivity and specificity; systolic blood pressure; time factor; time factors; triacylglycerol; tumor necrosis factor; waist circumference.

**Cluster 3 (75 items): Epidemiology / lifestyle determinants / risk factors & survival analysis**

adolescent; age; aged, 80 and over; alcohol consumption; body mass; body weight; breast neoplasms; breast tumor; caloric intake; cancer diagnosis; cancer grading; cancer incidence; cancer mortality; cancer prevention; cancer recurrence; cancer risk; cancer screening; cancer survivor; cancer survivors; case-control study; china; clinical trials; cohort analysis; cohort study; colorectal neoplasms; colorectal tumor; comparative study; controlled study; cross-sectional study; diet; dietary intake; disease-free survival; educational status; epidemiology; estrogen receptor; family history; follow-up study; food intake; health behavior; healthy lifestyle; lifestyle; lifestyle modification; longitudinal study; mediterranean diet; multicenter study; neoplasm recurrence; nutritional assessment; observational study; postmenopause; priority journal; proportional hazards; prospective study; psychology; questionnaire; risk factor; risk factors; risk reduction; sedentary lifestyle; self report; sex difference; smoking; statistics and numerical data; surveys and questionnaires; survival analysis; survival rate; survivorship; tumor recurrence; usa; very elderly; vitamin D; young adult.

**Cluster 4 (34 items): Tumor biology / molecular mechanisms & tumor microenvironment**

animal; apoptosis; biomarkers, tumor; cancer growth; carcinogenesis; cell proliferation; colon cancer; disease course; disease exacerbation; disease progression; gene expression; gene mutation; genetics; human cell; liver cell carcinoma; metabolism; metastatic; microrna; nonhuman; oxidative stress; personalized medicine; phenotype; prostate cancer; prostate tumor; prostatic neoplasms; protein expression; review; signal transduction; tumor growth; tumor invasion; tumor microenvironment; tumor volume; unclassified drug; upregulation.

**Cluster 5 (26 items): Body size/obesity & cancer types / outcomes / evidence synthesis terms**

association; body mass index; breast cancer; cancer; colorectal cancer; diagnosis; endometrium cancer; guidelines; health; impact; meta analysis; meta-analysis; mortality; nutrition; obesity; outcome; ovarian cancer; overweight; prevention; recurrence; risks; skeletal-muscle; survival; systematic review; weight loss; women.

**Appendix S2. Full search strategy (Web of Science Core Collection and Scopus)**

Note: Following reviewers’ suggestions, the main text provides a concise description of the search strategy; the complete Boolean search strings are reported here for transparency and reproducibility.

**S2.1 Web of Science Core Collection (SCI-EXPANDED)**

Database: Web of Science Core Collection (SCI-EXPANDED)

Fields: Topic (TS)

Timespan: 2015–2024

Language: English

Document types: Article OR Review

Search query (Boolean):

TS = (("exercise" OR "physical activity" OR "exercise training" OR training OR "aerobic exercise" OR "resistance training" OR "strength training" OR walking OR "rehabilitation" OR "prehabilitation") AND (cancer* OR neoplasm* OR tumor* OR tumour* OR carcinoma* OR malignan* OR "oncology") AND (prognos* OR "cancer survival" OR survival OR "overall survival" OR "disease-free survival" OR DFS OR "progression-free survival" OR PFS OR recurrence OR "treatment outcome*" OR "patient-reported outcome*" OR "quality of life"))

**S2.2 Scopus**

Database: Scopus

Fields: TITLE-ABS-KEY

Timespan: 2015–2024

Language: English

Document types: Article OR Review

Search query (Boolean):

TITLE-ABS-KEY (("exercise" OR "physical activity" OR "exercise training" OR training OR "aerobic exercise" OR "resistance training" OR "strength training" OR walking OR rehabilitation OR prehabilitation) AND (cancer* OR neoplasm* OR tumor* OR tumour* OR carcinoma* OR malignan* OR oncology) AND (prognos* OR "cancer survival" OR survival OR "overall survival" OR "disease-free survival" OR "progression-free survival" OR recurrence OR "treatment outcome*" OR "patient-reported outcome*" OR "quality of life"))
